# Cohort Profile: Migrant Health Follow-Up Study (MHFUS) of internal migration in South Africa

**DOI:** 10.1093/ije/dyae081

**Published:** 2024-06-12

**Authors:** Carren Ginsburg, Mark A Collinson, Chantel F Pheiffer, F Xavier Gómez-Olivé, Sadson Harawa, Stephen T McGarvey, Daniel Ohene-Kwofie, Andrew D Foster, Tyler W Myroniuk, Mark N Lurie, Stephen M Tollman, Michael J White

**Affiliations:** Medical Research Council/Wits Rural Public Health and Health Transitions Research Unit (Agincourt), School of Public Health, Faculty of Health Sciences, University of the Witwatersrand, Johannesburg, South Africa; Medical Research Council/Wits Rural Public Health and Health Transitions Research Unit (Agincourt), School of Public Health, Faculty of Health Sciences, University of the Witwatersrand, Johannesburg, South Africa; Department of Science and Innovation/Medical Research Council, South African Population Research Infrastructure Network, Durban, South Africa; Population Studies and Training Center, Brown University, Providence, RI, USA; Department of Urban Public Health, University of Massachusetts, Boston, MA, USA; Medical Research Council/Wits Rural Public Health and Health Transitions Research Unit (Agincourt), School of Public Health, Faculty of Health Sciences, University of the Witwatersrand, Johannesburg, South Africa; Medical Research Council/Wits Rural Public Health and Health Transitions Research Unit (Agincourt), School of Public Health, Faculty of Health Sciences, University of the Witwatersrand, Johannesburg, South Africa; Population Studies and Training Center, Brown University, Providence, RI, USA; Department of Epidemiology and International Health Institute, School of Public Health, Brown University, Providence, RI, USA; Department of Anthropology, Brown University, Providence, RI, USA; Medical Research Council/Wits Rural Public Health and Health Transitions Research Unit (Agincourt), School of Public Health, Faculty of Health Sciences, University of the Witwatersrand, Johannesburg, South Africa; Population Studies and Training Center, Brown University, Providence, RI, USA; Department of Public Health, University of Missouri, Columbia, MO, USA; Population Studies and Training Center, Brown University, Providence, RI, USA; Department of Epidemiology and International Health Institute, School of Public Health, Brown University, Providence, RI, USA; Medical Research Council/Wits Rural Public Health and Health Transitions Research Unit (Agincourt), School of Public Health, Faculty of Health Sciences, University of the Witwatersrand, Johannesburg, South Africa; Medical Research Council/Wits Rural Public Health and Health Transitions Research Unit (Agincourt), School of Public Health, Faculty of Health Sciences, University of the Witwatersrand, Johannesburg, South Africa; Population Studies and Training Center, Brown University, Providence, RI, USA

**Keywords:** Longitudinal study, cohort, internal migration, South Africa, health

Key Features

- The Migrant Health Follow-Up Study (MHFUS) was established to address a knowledge gap about the consequences of migration and urbanization for individual health in a dynamic socioeconomic transition setting in South Africa.
- The cohort is based on a simple random sample of 3800 18–40-year-olds selected from the Agincourt Health and socio-Demographic Surveillance System (HDSS) in 2017, and consists of both highly mobile internal migrants and Agincourt HDSS residents remaining in their rural origin.
- The cohort has thus far been followed for four study waves between 2018 and 2022, with information on education, employment, migration, household composition, health (including general health, chronic conditions, HIV, sleep), health service use and diet collected in all waves; biometric and anthropometric measures collected during the face-to-face interview rounds in Waves 1 and 4 (interviews were administered via telephone in Waves 2 and 3).
- The study has achieved exceptional rates of cohort retention, with 98% of Wave 1 participants re-interviewed in Wave 4 (*n* = 3039).
- The MHFUS is being conducted collaboratively between investigators at Brown University (USA) and the MRC/Wits Rural Public Health and Health Transitions Research Unit, University of the Witwatersrand (South Africa). Cohort data are published in [https://data.agincourt.co.za/index.php/catalog/395].

## Why was the cohort set up?

Changes in population distribution through migration and urbanization are associated with transitions in health exposure regimens and health outcomes.^1^ However, our understanding of these links remains limited. South Africa’s disease burden is among the highest of the world’s middle-income countries, with high prevalence of infectious disease (ID) and non-communicable disease (NCD),^2^^,^^3^ exacerbated by inadequate monitoring and availability of health information and services.^4^ Internal mobility in South Africa is high as people move to urban areas in search of work, often on a temporary basis. These patterns of mobility are rooted in the apartheid legacy of circular and temporary migration between rural and urban areas.^5–7^

The Migrant Health Follow-Up Study (MHFUS) responds to the need to fill this knowledge gap. The MHFUS, funded by the National Institutes of Health, USA, began recruitment in 2017 in the Bushbuckridge district, Mpumalanga Province, in South Africa’s northeast. We aim specifically to understand whether and how internal migration and urbanization affect NCD and ID risk, and whether migration compromises treatment continuity. By examining migration dynamics at high spatial and temporal resolutions, with attention to selection and heterogeneity, we aim to assess these health burdens and trace their concomitants in human behaviour amid dynamic sociodemographic change.

## Who is in the cohort?

The MHFUS is an observational cohort based on a sample of 18–40-year-olds who in 2016 were part of the population under demographic surveillance in the Agincourt Health and socio-Demographic Surveillance System (HDSS). The Agincourt HDSS study site, located in South Africa’s Mpumalanga Province, was established in 1992 and has a current population of approximately 115 000.^8^^,^^9^ This population includes individuals residing within the study site, as well as those residing outside the HDSS catchment area (migrants) who report connections to their HDSS household through visits and remittances. In 2016, 18–40-year-olds constituted 41.3% of the Agincourt HDSS population, with the prevalence of migration in this age range approximately 40% among females and 60% among males. Migration in young adults is most often motivated by job-seeking or accessing further education and may be short-term (temporary) or involve longer term relocation.^10^^,^^11^ In turn, these shifts in location and attendant lifestyle and socioeconomic conditions present important health considerations.

In 2017, we randomly selected 3800 individuals in the 18–40 age range to form the MHFUS cohort. Multiple sampled participants (within the eligible age range) were permitted per household—we had 3434 unique-origin households in the sample of 3800. The sample size enabled sufficient statistical power for analysis of migrant-nonmigrant differentials, controlling for key covariates such as age (within the cohort frame), sex and education. In addition to new data collection on the cohort, we are able to link cohort data to historical surveillance data on households and individuals that go back to the establishment of the Agincourt HDSS in 1992, facilitating analysis of participants prior health and individual and household circumstances.

Fieldwork commenced with initial household visits to the sampled individuals’ origin households within the Agincourt study site. MHFUS household visits located eligible participants if they resided within the Agincourt study site or retrieved their contact and address information if they were living elsewhere. (An earlier pilot study had confirmed the feasibility of contacting and interviewing migrants by telephone and in person.^12^)

**Figure 1.**
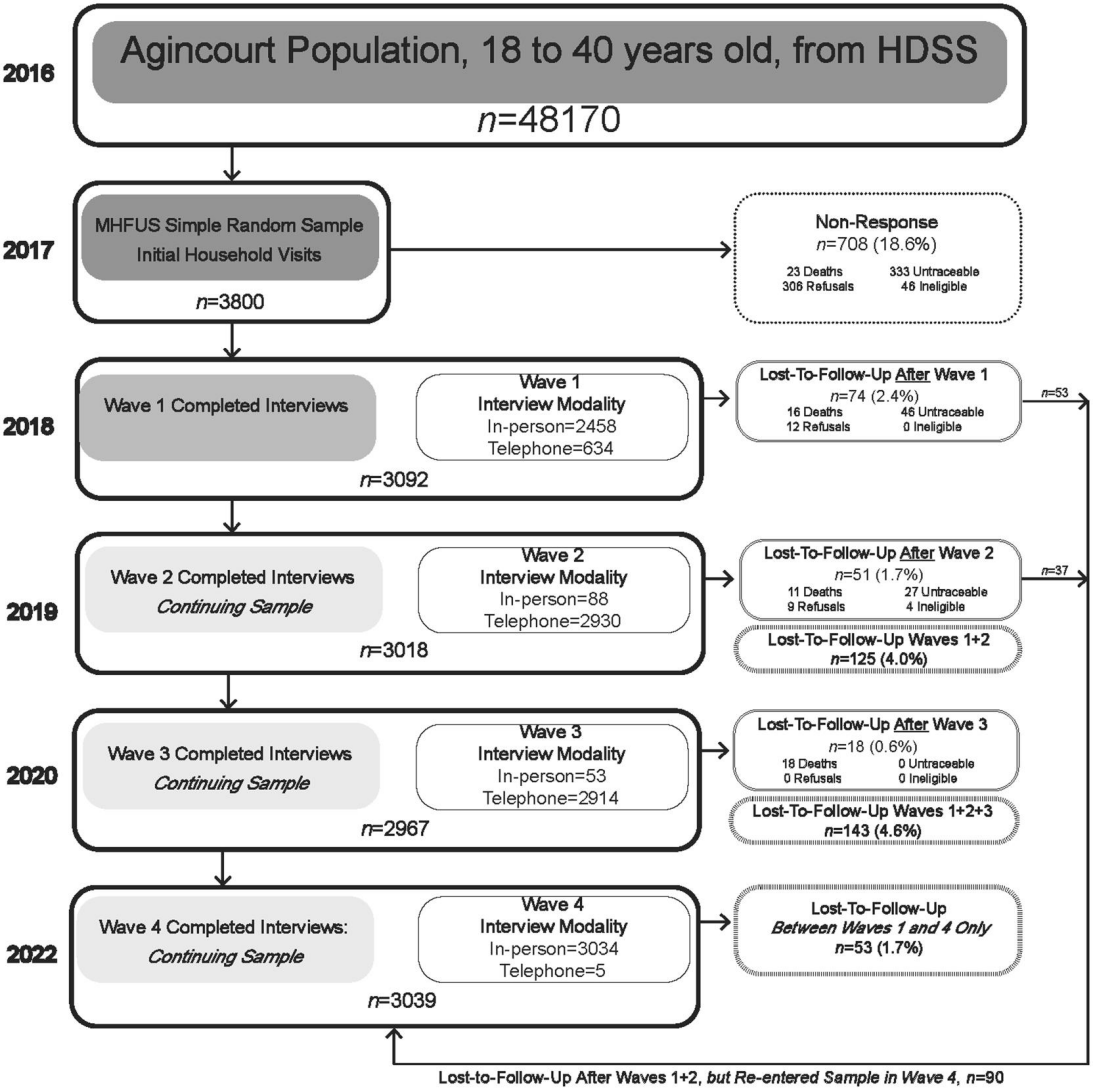
Flow chart of Migrant Health Follow-Up Study sample enrolment and retention

We successfully enrolled 81% (*n *=* *3092) of those sampled into the MHFUS study. Shortfall in enrolment was due to: inability to trace a participant; refusal by the household or individual; death; incapacitation; out-of-range age of the sampled individual; and the discovery of a few duplicate sampled HDSS records (see Figure 1). A comparison of the demographic characteristics of those enrolled versus not enrolled is presented in Table 1. Age and sex composition did not differ between the enrolled and non-enrolled. A larger proportion of those not enrolled had primary or lower levels of education (13.0%) compared with those enrolled (6.9%), originated from households with socioeconomic status in the lowest quintile (17.2% compared with 14.4%) and were migrants (53.4% compared with 42.2%) remaining attached to their origin households. (A person weight is available in our data repository to adjust for differential participation of sampled individuals in Wave 1.) In contrast to other surveillance-based studies, we continue to follow all enrolled members, irrespective of additional geographical mobility.

**Table 1.** Characteristics of Migrant Health Follow-Up Study participants who completed the baseline interview and those not enrolled

	Full cohort (*n *=* *3800)	Not enrolled (*n *=* *708)	Enrolled (*n *=* *3092)	
Characteristic	*n* (%)	*n* (%)	*n* (%)	***P*** ^a^
Age (years)				
Mean (SD)	28.3 (5.8)	28.6 (6.0)	28.2 (5.7)	0.103
Min, max	16, 42	16, 42	18, 40	
Sex				
Male	1918 (50.5%)	366 (51.7%)	1552 (50.2%)	0.471
Female	1882 (49.5%)	342 (48.3%)	1540 (49.8%)	
Education status				
Primary school or lower	306 (8.1%)	92 (13.0%)	214 (6.9%)	<0.001
High school incomplete	1619 (42.6%)	277 (39.1%)	1342 (43.4%)	
Matric or post school	1875 (49.3%)	339 (47.9%)	1536 (49.7%)	
Residence status				
Agincourt HDSS resident	2116 (55.7%)	330 (46.6%)	1786 (57.8%)	<0.001
Migrant	1684 (44.3%)	378 (53.4%)	1306 (42.2%)	
Socioeconomic status quintile^b^ (1 = poorest, 5 = richest)				
1	568 (14.9%)	122 (17.2%)	446 (14.4%)	<0.001
2	709 (18.7%)	119 (16.8%)	590 (19.1%)	
3	754 (19.8%)	147 (20.8%)	607 (19.6%)	
4	816 (21.5%)	137 (19.3%)	679 (22.0%)	
5	919 (24.2%)	167 (23.6%)	752 (24.3%)	
Missing	34 (0.9%)	16 (2.3%)	18 (0.6%)	
Marital status				
Single	2774 (73.0%)	485 (68.5%)	2289 (74.0%)	<0.001
Partnered	716 (18.8%)	120 (16.9%)	596 (19.3%)	
Missing	310 (8.2%)	103 (14.5%)	207 (6.7%)	

SD, standard deviation; min, minimum value; max, maximum value; HDSS, Health and socio-Demographic Surveillance System.

a *P-*values based on chi square or t tests.

b This measure was based on a wealth index constructed on the basis of household assets, and divided into quintiles (see^27^).

## How often have they been followed up?

Wave 1 fieldwork occurred between February 2018 and January 2019, by two teams. Migrant participants living in the Gauteng Province (a common migrant destination) were assigned to a Johannesburg-based fieldwork team who visited participants at their residences or workplaces. Participants residing within, or nearby, the Agincourt study site were assigned to the HDSS-based fieldwork team. In-person surveying was the primary modality for the interviews in Wave 1, although 20.5% (*n *=* *634) of the Wave 1 sample was interviewed via telephone because participants were difficult to reach in person (see Figure 1).

Wave 2 fieldwork occurred from September 2019 until January 2020, with a cohort retention rate of 97.6% between Waves 1 and 2. After Wave 1, 74 participants were lost to follow-up due to death, refusal, ineligibility, or inability to locate the participant. Wave 2 fieldwork was conducted primarily via cellular phone (although 88 participants requested an in-person interview), and 51 participants were subsequently lost to follow-up.

Wave 3 fieldwork took place between September 2020 and March 2021, with interviews conducted primarily over the phone as per protocol. We re-interviewed 2967 participants, retaining 96% of the cohort interviewed in Wave 1, with 18 participants lost to follow-up after Wave 3.

Wave 4 of data collection ran from January to September 2022, with interviews conducted in person by two fieldwork teams parallel to Wave 1. We reached 3034 participants in person and five participants telephonically. In addition, we located and re-interviewed 90 of the 143 participants who were lost to follow-up after Waves 1 and 2, for a final Wave 4 sample of 3039. Ultimate loss to follow-up was 53 participants (1.7%), 45 of whom had died during the study period. We retained 95.3% of the cohort for all four waves over the 2018–22 period. Wave 5 of the study is currently under way, and we plan to continue to follow the enrolled cohort as they move from younger to middle adult ages.

## What has been measured?

All four waves included a survey with questionnaire modules on education and employment, residential history, social conditions, health status and health behaviours (see Table 2 for details). We maintained consistency across questionnaire rounds using harmonized questions that allow for analysis of change over time. The questionnaire took on average 30 min to complete on tablets using REDCap electronic data capture software hosted at the University of the Witwatersrand.^13^^,^^14^

**Table 2.** Domains of measures investigated in the Migrant Health Follow-Up Study by study wave

	Wave 1	Wave 2	Wave 3	Wave 4
**Demographic characteristics**				
Age, sex	✓	✓	✓	✓
**Education and economic factors**				
Education—highest level completed	✓	✓	✓	✓
Currently engaged in education	✓	✓	✓	✓
Current employment status	✓	✓	✓	✓
Occupation	✓	✓	✓	✓
Hours of work	✓	✓	✓	✓
Income	✓	✓	✓	✓
Employment history	✓	✓	✓	✓
Remittance behaviour (value of money, goods sent home)	✓	✓	✓	✓
**Geography and migration** ^a^				
Origin village within the Agincourt HDSS^b^	✓	✓	✓	✓
Current place of residence (area and province)^c^	✓	✓	✓	✓
Duration of residence^d^	✓	✓	✓	✓
Urbanicity (area) and type of dwelling	✓	✓	✓	✓
Residence history (other places lived in for >6 months)	✓	✓	✓	✓
Reasons for move	✓	✓	✓	✓
Short-term absences (periods of >1 month in past year)	✓	✓	✓	✓
Frequency of visits to origin household	✓	✓	✓	✓
Transport use and safety		✓	✓	✓
**Social factors**				
Household size/composition	✓	✓	✓	✓
Household roster	✓	✓	✓	✓
Relation to household head	✓	✓	✓	✓
Communication with origin household (frequency and mode)	✓	✓	✓	✓
Social capital (networks and support)	✓	✓	✓	✓
Maternity and paternity history (number of children, ages, residential arrangements)		✓	✓	✓
Mode of transportation and safety		✓	✓	✓
COVID—impact on earnings, social relief grant, knowledge, behaviours			✓	✓
**Health**				
Self-reported health status	✓	✓	✓	✓
Diagnosis of chronic condition, place and time of diagnosis, receiving treatment	✓	✓	✓	✓
Use of health services (type of service, location of facility)	✓	✓	✓	✓
Medical aid/health insurance		✓	✓	✓
HIV (self-reported status, testing, antiretroviral therapy use)	✓	✓	✓	✓
Mental health: 10-item Center for Epidemiological Studies Depression Scale		✓	✓	✓
Grit scale		✓	✓	✓
Pregnancy status	✓	✓	✓	✓
COVID vaccination, infection			✓	✓
**Health behaviours**				
Food security and diet	✓	✓	✓	✓
Tobacco and alcohol use	✓	✓	✓	✓
Sedentary behaviour and sleep	✓	✓	✓	✓
Pittsburgh Sleep Quality Index Questionnaire		✓	✓	✓
Sexual partnerships (number of partners, location of partners, condom use, HIV status)	✓	✓	✓	✓
**Physical measurements**				
Blood pressure (systolic, diastolic)^e^	✓			✓
Pulse^e^	✓			✓
Height^f^	✓			✓
Weight^f^	✓			✓
Waist circumference^g^	✓			✓
HIV status, viral load^h^	✓			✓
Glycated haemoglobin (HbA1c)^h^	✓			✓

a Using the Health and socio-Demographic Surveillance System (HDSS) study site boundary as a relevant geographical feature for defining migration, cohort members may be classified, in the cross section, either as residents of the Agincourt study site (non-migrants) or migrants living outside. The Migrant Health Follow-Up Study (MHFUS) additionally captures the timing of movement and is able to classify individuals across waves as ‘out-migrants’ (moving out of the HDSS study site), ‘return migrants’ (returning back to the HDSS study site after a period living away) or ‘circular migrants’ (moving multiple times between the HDSS study site and destinations outside of the HDSS study site).

b The Agincourt HDSS study site is a 420 km^2^ area comprising 31 contiguous villages from which the MHFUS cohort was randomly selected. All cohort participants have their origin in one of these villages.

c An individual’s current place of residence refers to the area and province in which a participant is residing at the time of the survey. This may be within the Agincourt HDSS study site, or outside the study site.

d The MHFUS, in keeping with many migration studies, reflect both space and time delimitations in capturing movements. The timing of movement and duration spent in a particular location are collected.

e Blood pressure (BP) and pulse rates were measured with an Omron HEM907 XL digital BP monitor. High blood pressure was defined as an average diastolic BP ≥90 mmHg or systolic BP ≥140 mmHg or self-report of antihypertensive treatment.

f Height was measured using an Omron stadiometer. Weight was measured using a bathroom scale on a level surface (QE-2008 in Wave 1 and Omron Body scale in Wave 4). Body mass index was classified as overweight, 25–29.9 kg/m^2^, or obese >30 kg/m^2^.

g Waist circumference was measured at the umbilicus level with a flexible plastic tape.

h HIV status, viral load, and glycated haemoglobin (HbA1c) were determined using dried blood spots (DBS) assays. DBS were collected using Whatman 903 TM filter paper, and packaged and frozen at -80°C.

In Wave 2, we added modules on depression risk,^15^ personal resilience/grit,^16^ sleep quality^17^ and a maternity/paternity history that documented all children and their places of residence. In Waves 3 and 4, with the emergence of the COVID-19 pandemic, we introduced targeted questions about the pandemic’s economic impacts, health-related behaviours and COVID-19 vaccine uptake.^18^

During the in-person data collection waves (1 and 4), anthropometric and blood pressure (BP) measurements were taken by locally trained fieldworkers. Weight and height, used to calculate body mass index (BMI), were measured using a bathroom scale and stadiometer (see Table 2). BP was measured three times in the seated position, using an appropriately sized upper-arm inflation cuff after an initial 5-min rest period, with 2 min of rest between the measurements. Five dried blood spots (DBS) were collected and used to test for HIV status, viral load and glycated haemoglobin (HbA1c).

Informed consent for participation in the study was received in writing from all participants who were interviewed face to face and verbally for interviews conducted telephonically. Results of the anthropometric measurements were provided to participants at the time of the interview. Participants who consented to HIV testing were offered the opportunity to receive their HIV status through a rapid HIV test administered in a follow-up visit from a trained health professional, or later in the study, through provision of an Oraquick HIV self-test given to participants at the time of the interview. In the case of HbA1c, results were confidentially communicated to participants by telephone once they became available following laboratory analysis.

## What has it found?

### Migration and geographies

**Figure 2.**
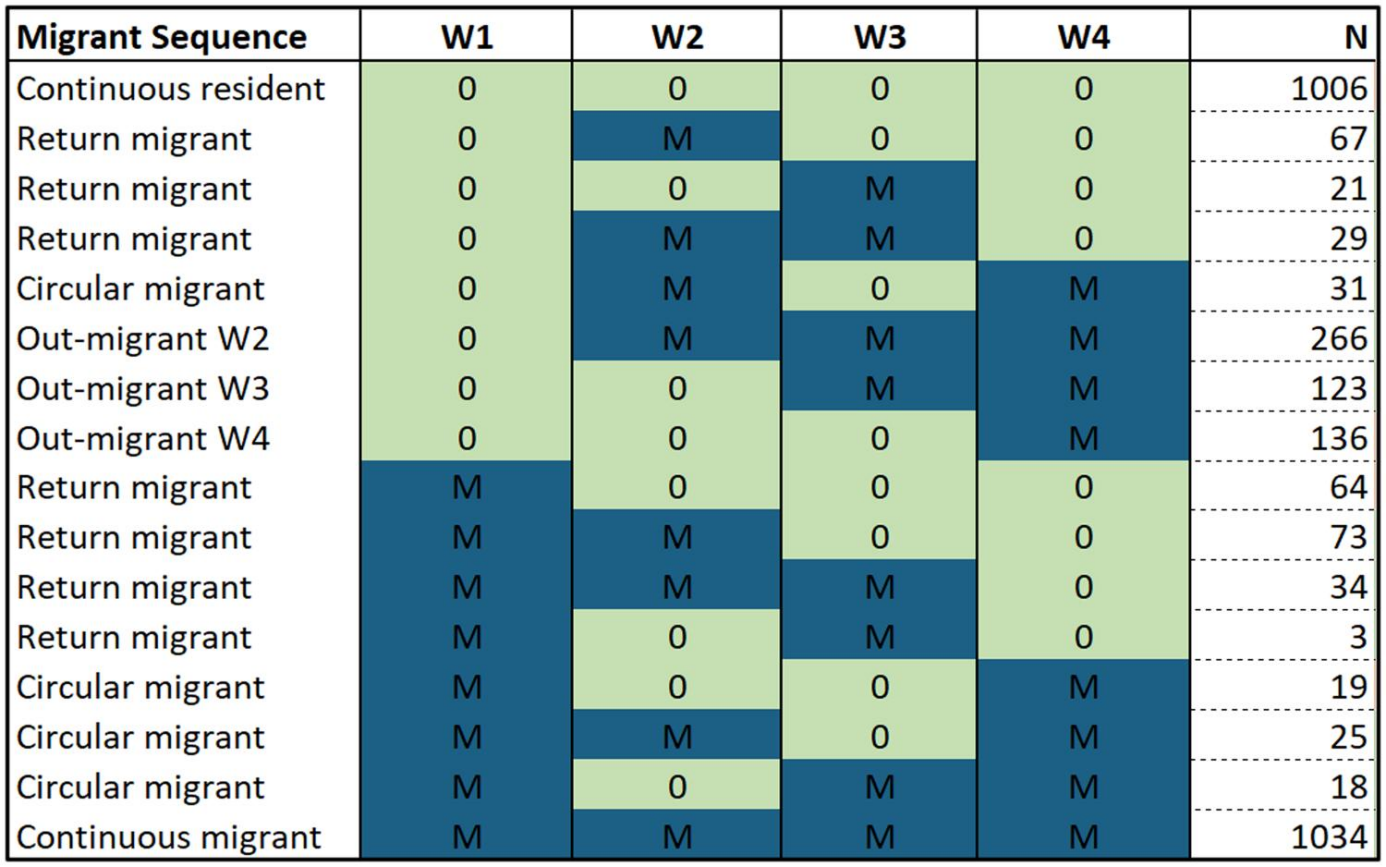
Migration sequences for continuous participants in the Migrant Health Follow-Up Study, Waves 1 to 4 (*n *=* *2949). M, migrant; 0, Agincourt Health and socio-Demographic Surveillance System resident

Among those interviewed in all four waves (*n *=* *2949), the share of migrants in the cohort were 43.1% in 2018 (Wave 1), 52.9% in 2019 (Wave 2), 51.8% in 2020 (Wave 3) and 56% in 2022 (Wave 4). A greater proportion of migrants were male, and migrants were more likely to have a completed high school or attained post-secondary education.^18–20^ We have observed a range of migration sequences over time (see Figure 2): 34% of the cohort lived continuously in the Agincourt study site, whereas 35% were continuous migrants (living in locations outside the Agincourt study site from Waves 1 to 4). Almost one-fifth of the sample (18%) experienced an out-migration over the period, 10% were return migrants and 3% were circular migrants. In all waves, most migrants were either living in the Gauteng Province (Wave 1: 50.9%; Wave 2: 45.8%; Wave 3: 43.5%; Wave 4: 43.3%) or in areas near the Agincourt study site and within the Mpumalanga Province (Wave 1: 38.9%; Wave 2: 41.9%; Wave 3: 44.2%; Wave 4: 45.6%).

### Socioeconomic and social conditions

The MHFUS has emphasized the collection of social and economic information in parallel with health measures. Much migration is prompted by the search for employment, and return migration is often associated with job loss.^18^ Levels of unemployment in the MHFUS have fluctuated among Agincourt residents over the study period (Wave 1: 46.3%; Wave 2: 59.3%; Wave 3: 65.2%; Wave 4: 53%), reflecting economic conditions and the COVID-19 pandemic. An analysis exploring the impact of COVID-19 revealed that 40.8% of Agincourt residents (disproportionately women) employed in Wave 2 were unemployed in Wave 3, whereas only 20.2% of return migrants continued employment between Waves 2 and 3.^18^ In addition, 21.3% of Agincourt residents and 11.3% of migrants reported food insecurity in Wave 3 (*P *<* *0.001) compared with 15.6% of residents and 4.1% of migrants in Wave 2.^18^

### Health status and use of health services

In this young adult cohort, a key objective is to determine how mobility impacts on risk factors for poor health and access to treatment. In line with the theory of positive health selection (the ‘heathy migrant hypothesis’),^1^^,^^21^ we found migrants to be in better health than Agincourt residents in Wave 1.^19^^,^^22^ In subsequent waves of the study, we found migrants to be at lower risk of depression and to display higher levels of grit (unpublished data).

**Figure 3.**
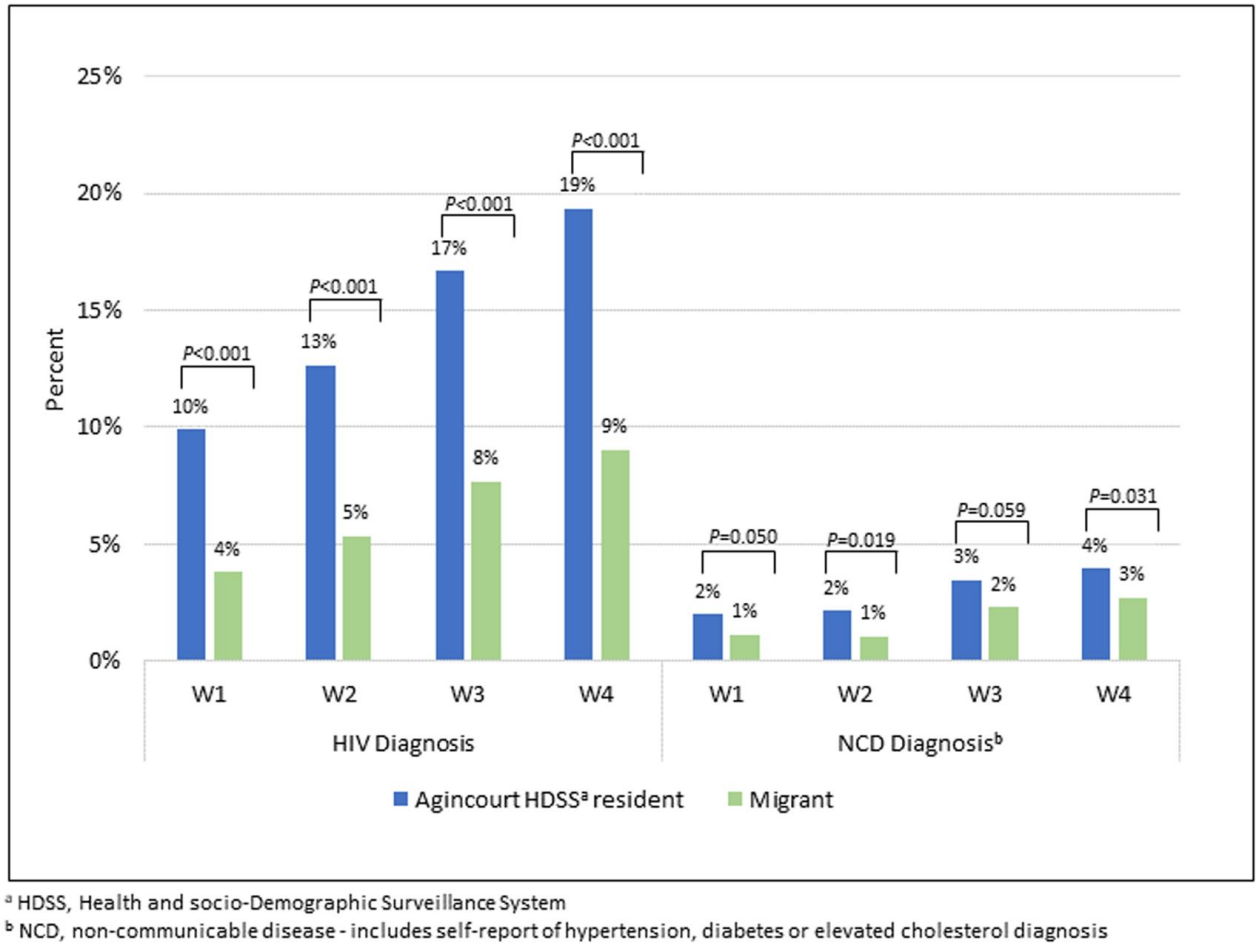
Self-reported diagnosis of a chronic condition for continuous participants in the Migrant Health Follow-Up Study, Waves 1 to 4 (*n *=* *2949)

The prevalence of chronic conditions was relatively low due to participants’ younger ages at baseline. We observed increases in self-reports of HIV-positive status and NCDs (hypertension, diabetes and elevated cholesterol) over time, with noticeable health differences favouring migrants (Figure 3). Notably, self-report of positive HIV status was lower than measured HIV-positive status, but self-report was predictive of true status for both migrants and Agincourt residents in Wave 1.^23^ The prevalence of other self-reported chronic conditions was also lower than those obtained from anthropometric measurements and biomarkers, which suggests relatively low knowledge about, and diagnosis of, chronic conditions in this population.^22^

Analysis of anthropometrics and BP have highlighted a health disadvantage—particularly among migrant women. Female migrants who spent all four waves living outside the Agincourt study site had 1.8 times the odds (95% CI: 1.2, 2.5) of having overweight and obesity than women remaining in Agincourt (after adjusting for diet, diabetes/hypertension, HIV status and baseline BMI). There were no such differences by migrant status among males (unpublished data). Similarly, whereas there were no significant differences in BP between male migrants and Agincourt residents, female migrants had an average 4.3-mmHg greater diastolic BP (adjusting for BMI and other health and socioeconomic characteristics) compared with Agincourt residents in Wave 1.^20^ Preliminary analysis of follow-up BP measurements collected in Wave 4 suggests that this finding persists over time. Gender and migration status emerge as important social determinants of health, and our data measure nuance in a way that few studies can.

Health service use differed by migrant status and by sex. At baseline, 71.0% of women and only 33.1% of men had made use of health services in the past year, and Agincourt residents were more likely than migrants to have used health services.^19^ Levels of health service use decreased between the study’s second and third waves (likely a consequence of the COVID-19 pandemic), with 56.6% of Agincourt residents and 51.1% of migrants reporting having accessed a health service in the past year in Wave 3 compared with 62.2% and 53.6% in Wave 2.^18^

A full listing of MHFUS publications can be found at https://sites.brown.edu/migration-and-health/].

## What are the main strengths and weaknesses?

Migrants are notoriously difficult to recruit and follow. Furthermore, selectivity of migration along demographic, socioeconomic and health dimensions means that simple comparisons of rural and urban populations may not reveal underlying differences ‘all else equal’. The MHFUS cohort’s unique study design makes for superior inferences in comparisons between movers and stayers in the study of urbanization and health. The MHFUS survey integrates the social and biological dimensions that are important for understanding the multifaceted nature of migration and corresponding environmental change, while also providing an opportunity to address concerns about the social determinants of health.^24–26^ In addition to self-reported health measures, we have collected objective measures of HIV, BP, glycated haemoglobin, BMI and central adiposity.

The study has achieved high sample retention for a mobile cohort, facilitated by the strong connection to participants’ origin communities. The greatest attrition took place at the study enrolment phase. Through experience, the team has improved retention methods and strategies in the field, resulting in increased in-person contacts between the first face-to-face round and the recent fourth wave of data collection. The study is not nationally representative, but it does demonstrate the feasibility of collection of such nationally representative data.

We experienced some challenges due to the geographical dispersion of participants; yet our success with telephone interviews point to the broader feasibility of interviewing geographically hard-to-reach participants. Ascertaining, recording and analysing geography consistently nevertheless remains a resource-intensive activity. The challenges presented by COVID-19 meant some delays in study time lines, but we overcame these with telephone surveys, while abiding by all appropriate health and safety protocols.

## Can I get hold of the data? Where can I find out more?

Data from Wave 1 of the Migrant Health Follow-Up Study are available through the MRC/Wits Agincourt Data repository [https://data.agincourt.co.za/index.php/catalog/395]. Data on the further study waves will be added to the repository in future releases, and specific data requests can be made to the corresponding author, Carren Ginsburg [carren.ginsburg@wits.ac.za]. Agincourt HDSS data are available through the South African Population Research Infrastructure Network (SAPRIN) data repository [https://saprindata.samrc.ac.za/index.php/catalog].

## Ethics approval

The Migrant Health Follow-Up Study was reviewed, and ethics approval received from the University of the Witwatersrand Human Research Ethics Committee (Medical) (clearance certificate numbers M170277 and M220160) and the Mpumalanga Province Department of Health Research and Ethics Committee.

## Data Availability

See ‘Can I get hold of the data?’ Above.

## References

[1] Nauman E , VanLandingham M, Anglewicz P, Migration, urbanization and health. In: White MJ (ed). International Handbook of Migration and Population Distribution. Dordrecht: Springer, 2016, pp. 451–64.

[2] Achoki T , Sartorius B, Watkins D et al Health trends, inequalities and opportunities in South Africa’s provinces, 1990-2019: findings from the Global Burden of Disease 2019 Study. J Epidemiol Community Health 2022;76:471–81. 35046100 10.1136/jech-2021-217480 PMC8995905

[3] Mayosi BM , Flisher AJ, Lalloo UG, Sitas F, Tollman SM, Bradshaw D. The burden of non-communicable diseases in South Africa. Lancet 2009;374:934–47. 19709736 10.1016/S0140-6736(09)61087-4

[4] Mayosi BM , Lawn JE, van Niekerk A, Bradshaw D, Abdool Karim SS, Coovadia HM; Lancet South Africa Team. Health in South Africa: changes and challenges since 2009. Lancet 2012;380:2029–43. 23201214 10.1016/S0140-6736(12)61814-5

[5] Posel D , Measuring labour migration after apartheid: patterns and trends. In: Bank LJ, Posel D, Wilson F (eds). Migrant Labour after Apartheid: The inside Story. Cape Town: HSRC Press, 2020, pp. 29–43.

[6] Wentzel M , Tlabela K, Historical background to South African migration. In: Kok P, Gelderblom D, Oucho JO, Van Zyl J (eds). Migration in South and Southern Africa: dynamics and Determinants. Cape Town: HSRC Press, 2006, pp. 71–96.

[7] Lurie MN , Williams BG. Migration and health in Southern Africa: 100 years and still circulating. Health Psychol Behav Med 2014;2:34–40. 24653964 10.1080/21642850.2013.866898 PMC3956074

[8] Kahn K , Collinson MA, Gómez-Olivé FX et al Profile: Agincourt Health and socio-Demographic Surveillance System. Int J Epidemiol 2012;41:988–1001. 22933647 10.1093/ije/dys115 PMC3429877

[9] Collinson MA , Mudzana T, Mutevedzi T et al Cohort profile: South African Population Research Infrastructure Network (SAPRIN). Int J Epidemiol 2021;51:e206–16. 10.1093/ije/dyab261 PMC9365637 34966919

[10] Ginsburg C , Collinson MA, Iturralde D et al Migration and settlement change in South Africa: triangulating Census 2011 with longitudinal data from the Agincourt Health and Demographic Surveillance System in the rural north-east. South Afr J Demogr 2016;17:133–98.

[11] Collinson M , Tollman S, Kahn K, Clark S, Garenne M, Highly prevalent circular migration: households, mobility and economic status in rural South Africa. In: Tienda M, Findley S, Tollman S, Preston-Whyte E (eds). Africa on the Move: African Migration and Urbanisation in Comparative Perspective. Johannesburg: Wits University Press, 2006, pp. 194–216.

[12] Pheiffer CF , McGarvey ST, Ginsburg C et al Dimensions of internal migration and their relationship to blood pressure in South Africa. J Biosoc Sci 2019;51:827–42. 31131777 10.1017/S0021932019000130 PMC6825752

[13] Harris PA , Taylor R, Minor BL et al The REDCap consortium: building an international community of software platform partners. J Biomed Inform 2019;95:103208. 31078660 10.1016/j.jbi.2019.103208 PMC7254481

[14] Harris PA , Taylor R, Thielke R, Payne J, Gonzalez N, Conde JG. Research electronic data capture (REDCap)—a metadata-driven methodology and workflow process for providing translational research informatics support. J Biomed Inform 2009;42:377–81. 18929686 10.1016/j.jbi.2008.08.010 PMC2700030

[15] Radloff LS. The CES-D Scale: a self-report depression scale for research in the general population. Appl Psychol Meas 1977;1:385–401.

[16] Duckworth AL , Quinn PD. Development and validation of the Short Grit Scale (Grit–S). J Pers Assess 2009;91:166–74. 19205937 10.1080/00223890802634290

[17] Buysse DJ , Reynolds CF 3rd, Monk TH, Berman SR, Kupfer DJ. The Pittsburgh Sleep Quality Index: a new instrument for psychiatric practice and research. Psychiatry Res 1989;28:193–213. 2748771 10.1016/0165-1781(89)90047-4

[18] Ginsburg C , Collinson MA, Gómez-Olivé FX, Harawa S, Pheiffer CF, White MJ. The impact of COVID-19 on a cohort of origin residents and internal migrants from South Africa’s rural northeast. SSM Popul Health 2022;17:101049. 35252532 10.1016/j.ssmph.2022.101049 PMC8889408

[19] Ginsburg C , Collinson MA, Gómez-Olivé FX et al Internal migration and health in South Africa: determinants of healthcare utilisation in a young adult cohort. BMC Public Health 2021;21:554. 33743663 10.1186/s12889-021-10590-6 PMC7981972

[20] Pheiffer CF , McGarvey ST, Ginsburg C, White MJ. Urban residence and elevated blood pressure among migrant women in South Africa. Health Place 2023;83:103071. 37421693 10.1016/j.healthplace.2023.103071 PMC10528937

[21] Anglewicz P , VanLandingham M, Manda-Taylor L, Kohler HP. Health selection, migration, and HIV infection in Malawi. Demography 2018;55:979–1007. 29704193 10.1007/s13524-018-0668-5 PMC5993628

[22] Dzomba A , Ginsburg C, Kabudula CW et al Epidemiology of chronic multimorbidity and temporary migration in a rural South African community in health transition: a cross-sectional population-based analysis. Front Epidemiol 2023;3:1054108. 38455922 10.3389/fepid.2023.1054108 PMC10910947

[23] Yorlets RR , Lurie MN, Ginsburg C et al Validity of self-report for ascertaining HIV status among circular migrants and permanent residents in South Africa: a cross-sectional, population-based analysis. AIDS Behav 2023;27:919–27. 36112260 10.1007/s10461-022-03828-w PMC9974592

[24] Marmot M , Friel S, Bell R, Houweling TA, Taylor S. Closing the gap in a generation: health equity through action on the social determinants of health. Lancet 2008;372:1661–69. 18994664 10.1016/S0140-6736(08)61690-6

[25] United States Department of Health and Human Services. *Healthy People 2030*. Washington, DC: Office of Disease Prevention and Health Promotion, 2023. https://health.gov/healthypeople/priority-areas/social-determinants-health (15 November 2023, date last accessed).

[26] Office of Behavioral and Social Sciences Research (OBSSR). *The Office of Behavioral and Social Sciences Research: Strategic Plan 2017–2021*. Maryland: OBSSR, 2016. https://obssr.od.nih.gov/sites/obssr/files/OBSSR-SP-2017-2021.pdf (15 November 2023, date last accessed).

[27] Kabudula CW , Houle B, Collinson MA et al Socioeconomic differences in mortality in the antiretroviral therapy era in Agincourt, rural South Africa, 2001-13: a population surveillance analysis. Lancet Glob Health 2017;5:e924–35. 28807190 10.1016/S2214-109X(17)30297-8 PMC5559644

